# Assessment of Antioxidant and Antibacterial Properties on Meat Homogenates of Essential Oils Obtained from Four *Thymus* Species Achieved from Organic Growth

**DOI:** 10.3390/foods6080059

**Published:** 2017-07-28

**Authors:** Carmen Ballester-Costa, Esther Sendra, Juana Fernández-López, Jose A. Pérez-Álvarez, Manuel Viuda-Martos

**Affiliations:** IPOA Research Group (UMH-1 and REVIV-Generalitat Valenciana), AgroFood Technology Department, Escuela Politécnica Superior de Orihuela, Miguel Hernández University, Ctra. Beniel km. 3,2., E-03312 Orihuela, Spain; carmen.ballester@umh.es (C.B.-C.); esther.sendra@umh.es (E.S.); j.fernandez@umh.es (J.F.-L.); ja.perez@umh.es (J.A.P.-A.)

**Keywords:** essential oil, *Thymus*, antibacterial, antioxidant, meat homogenates

## Abstract

In the organic food industry, no chemical additives can be used to prevent microbial spoilage. As a consequence, the essential oils (EOs) obtained from organic aromatic herbs and spices are gaining interest for their potential as preservatives. The organic *Thymus zygis*, *Thymus mastichina*, *Thymus capitatus* and *Thymus vulgaris* EOs, which are common in Spain and widely used in the meat industry, could be used as antibacterial agents in food preservation. The aims of this study were to determine (i) the antibacterial activity using, as culture medium, extracts from meat homogenates (minced beef, cooked ham or dry-cured sausage); and (ii) the antioxidant properties of organic EOs obtained from *T. zygis*, *T. mastichina*, *T. capitatus* and *T. vulgaris*. The antioxidant activity was determined using different methodologies, such as Ferrous ion-chelating ability assay, Ferric reducing antioxidant power, ABTS radical cation (ABTS^•^+) scavenging activity assay and 2,2′-diphenyl-1-picrylhydrazyl (DPPH) radical scavenging method; while the antibacterial activity was determined against 10 bacteria using the agar diffusion method in different meat model media. All EOs analyzed, at all concentrations, showed antioxidant activity. *T. capitatus* and *T. zygis* EOs were the most active. The IC_50_ values, for DPPH, ABTS and FIC assays were 0.60, 1.41 and 4.44 mg/mL, respectively, for *T. capitatus* whilst for *T. zygis* were 0.90, 2.07 and 4.95 mg/mL, respectively. Regarding antibacterial activity, *T. zygis* and *T. capitatus* EOs, in all culture media, had the highest inhibition halos against all tested bacteria. In general terms, the antibacterial activity of all EOs assayed was higher in the medium made with minced beef than with the medium elaborated with cooked ham or dry-cured sausage.

## 1. Introduction

Greater understanding of the relationship between diet, specific food ingredients and health is leading to new insights into the effect of food components on physiological function and health. This awareness has moved consumers to become more health-conscious, driving a trend towards “green”, healthy and nutritious foods with additional health-promoting functions. This new approach to improving health status is especially interesting for the meat industry. The study by Grunert [1] on trends in meat consumption identifies the processed meat manufacturing sector as having the most promising future, due, among other reasons, to consumers’ demand for products that are easy and quick to prepare. However, to maintain the safety and prolong the shelf-life of meat and meat products, the meat industry uses synthetic preservatives that have been widely used to control the lipid oxidation and to eliminate bacteria or moulds.

The use of these synthetic preservatives enters into controversy with the idea of a healthy and “green” product due to the fact that these compounds could cause health problems for consumers over a long-term period. Thus, aiming at the reduction of the use of chemical additives in the food industry, there has been growing interest recently in the use of natural food additives with antimicrobial and antioxidant properties that do not have any negative effects on human health [2]. In this way, natural antioxidants extracted from plants can be used as alternatives to synthetic preservatives due to their equivalent or greater effect on the inhibition of lipid oxidation and bacterial growth [3]. 

Essential oils (EOs) obtained from aromatic herbs and spices are aromatic oily liquids formed by aromatic plants as secondary metabolites, which are constituted by a complex mix of compounds, including monoterpens and sesquiterpene hydrocarbons, as well as their corresponding oxidized products (e.g., alcohols, aldehydes, ethers and ketones), several phenylpropane derivatives, phenols and miscellaneous volatile organic compounds (e.g., octanal, dodecanal, 2-undecanone) [4,5]. Although the antioxidant and antimicrobial properties of EOs were acknowledged a long time ago, there are still several investigations that have shown that these compounds exhibit strong antimicrobial and antioxidant properties [6,7,8], making them interesting ingredients in the meat industry. Additionally, the main advantage of EOs is that they can be used in any food, and are generally recognized as safe (GRAS), as long as their maximum effects are attained with minimal change in the organoleptic properties of the food [9]. Although the antimicrobial properties of EOs reaches in vitro bioactive concentrations at 5% or less, the application of plant EOs for control of food-borne pathogens and food spoilage bacteria requires the evaluation of their efficacy within food products or in model systems that closely simulate food composition [10]. The organic essential oils were obtained from four *Thymus* species: *Thymus zygis*, *Thymus mastichina*, *Thymus capitatus* and *Thymus vulgaris* chemotype linalool, which are common in Spain and are widely used in the meat industry. Additionally, they are widely used as culinary flavoring agents, and their flavor and aroma are familiar to and widely accepted by consumers [11]. Therefore, the aims of this study were to determine (i) their antibacterial activity using, as culture medium, extracts from meat homogenates (minced beef, cooked Ham or dry-cured sausage); and (ii) their antioxidant properties. 

## 2. Material and Methods

### 2.1. Essential Oils

The essential oils (EOs) of *Thymus zygis* reference (ref.) 11961, *Thymus mastichina* ref. 90001-1284, *Thymus capitatus* ref. 95001-1150, and *Thymus vulgaris* ref. 80001-3577 were used in this work. These EOs were analyzed by Ballester-Costa et al. [11]. These authors reported that in *T. mastichina* EO the major compounds were 1,8-cineole (51.94%), linalool (19.90%) and β-pinene (3.39%). *T. capitatus* EO was characterized by the high monoterpenoid fraction, and especially by the presence of carvacrol (69.83%), and their precursors *p*-cymene (6.12%) and γ-terpinene (6.68%). With regard to *T. vulgaris* EO, the main component was linalool (44.00%) followed by terpineol-4 (11.84%), γ-terpinene (8.91%) and β-myrcene (6.89%). Finally, in *T. zygis* EO, the major components were thymol (48.59%), *p*-cymene (18.79%), γ-terpinene (8.31%) and linalool (4.31%). All EOs analyzed were supplied by Esencias Martinez Lozano (Murcia, Spain). The EOs were certified organic by the Institute for Marketecology (IMO) according to the procedures as outlined in the USDA, AMS 7 CFR Part 205 National Organic Program, Final Rule.

### 2.2. Antioxidant Activity

#### 2.2.1. 2,2′-diphenyl-1-picrylhydrazyl (DPPH) Radical Scavenging Method

The antioxidant activity of different concentrations (0.23–30 mg/mL) of *Thymus* EOs was measured in terms of hydrogen donating or radical scavenging ability, using the stable radical DPPH [12]. The results were expressed as IC_50_ value: concentration (mg/mL) for a 50% chelating effect. 

#### 2.2.2. ABTS Radical Cation (ABTS^•+^) Scavenging Activity Assay

The ABTS^•+^ scavenging activity assay was determined as described by Leite et al. [13] with some modifications. The ABTS^•+^ solution was produced by reacting aqueous ABTS solution (7 mM) with potassium persulfate (2.45 mM). Diluted ABTS^•+^ solution with an absorbance of 0.70 ± 0.02 at 734 nm was employed in the analysis. The reactions were performed by adding 990 μL of ABTS^•+^ solution to 10 μL of each EOs solution (0.23–30 mg/mL). After 6 min of incubation at room temperature, absorbance values were measured on a spectrophotometer at 734 nm. The results were expressed as IC_50_ value: concentration (mg/mL) for a 50% chelating effect. 

#### 2.2.3. Ferric Reducing Antioxidant Power 

The ferric reducing antioxidant power (FRAP) of different concentrations (0.23–30 mg/mL) of *Thymus* EOs samples was determined by using the potassium ferricyanide-ferric chloride method [14]. The FRAP of the samples was estimated in terms of mg Trolox equivalent (TE) mL of the sample as the mean of three replicates.

#### 2.2.4. Ferrous Ion-Chelating Ability Assay

Ferrous ion (Fe^2+^) chelating activity (FIC) of different concentrations (0.15–20 mg/mL) of EO samples was measured by inhibiting the formation of Fe^2+^-ferrozine complex after treatment of test material with Fe^2+^, following the method of Carter [15]. The results were expressed as IC_50_ value: concentration (mg/mL) for a 50% chelating effect. 

### 2.3. Microbial Strains

The EOs were individually tested against several bacterial strains: *Listeria innocua* CECT 910, *Serratia marcescens* CECT 854, *Pseudomonas fragi* CECT 446, *Pseudomonas fluorescens* CECT 844, *Aeromonas hydrophila* CECT 5734, *Shewanella putrefaciens* CECT 5346, *Achromobacter denitrificans* CECT 449, *Enterobacter amnigenus* CECT 4078, *Enterobacter gergoviae* CECT 587, *Alcaligenes faecalis* CECT 145. These microorganisms were chosen as they are commonly associated with the spoilage of refrigerated foods; as an indicator of pathogenic microorganism or as the spoilage microorganism. All species were supplied by the Spanish Type Culture Collection (CECT) of the University of Valencia (Valencia, Spain). 

### 2.4. Antimicrobial Screening

#### 2.4.1. Preparation of Meat Model Medium

Ten grams of minced beef (MB), cooked ham (CH), or dry-cured sausage (DCS) were added to 90 mL of one-quarter-strength buffered peptone water (pH 7.2) in blender bags and homogenized in a Stomacher until smooth. After that, the samples were filtered through a paper disc Whatman n° 2 to remove solid particles and obtained a clarified extract. Meat model medium was made mixing the extracts obtained from MB, CH or DCS with agar solution (Sharlab, Barcelona, Spain) in order to obtain a final solid medium solution with 1.5% agar. Finally, all prepared meat solutions were autoclaved, separately, at 121 °C for 15 min prior to use, to eliminate contamination from organisms that may already be present in the food. 

#### 2.4.2. Disc-Diffusion Method

Screening of EOs for antibacterial activity was determined by the agar diffusion method following the recommendations of Tepe et al. [16]. Petri plates were prepared by pouring 20 mL of previously prepared meat model medium (MB, CH or DCS) at 55 °C and allowed to solidify. Plates were dried for 30 min in a biological safety cabinet with vertical laminar flow. A suspension (0.1 mL of 10^6^ CFU/mL) of standardized inoculum suspension was spread on the solid medium plates. The inoculums were allowed to dry for 5 min. Then, a sterile filter paper disk (9 mm in diameter Schlinder & Schuell, Dassel, Germany) was impregnated with 30 μL EO. The plates were left for 15 min at room temperature to allow the diffusion of the EO, and then they were incubated at appropriated temperature for each bacterium for 24 h. At the end of the period, the diameter of the clear zone around the disc was measured with a caliper (Wiha dialMax^®^ ESD-Uhrmessschieber) and expressed in millimeters (disk diameter included) as its antimicrobial activity. According to the width of the inhibition zone diameter expressed in mm, results were appreciated as follows: not active (−) for diameters equal to or below 12.0 mm; moderately active (+) for diameters between 12.0 and 21.0 mm; active (++) for diameters between 21.0 and 30.0 mm and extremely active (+++) for diameters equal to or longer than 30.0 mm [17]. All tests were performed in triplicate. 

### 2.5. Statistical Analysis

Conventional statistical methods were used to calculate means and standard deviations of three simultaneous assays carried out with the different methods. Data collected for antioxidant properties were analyzed by one-way analysis of variance to test the effects of essential oils (levels: *T. zygis*, *T. mastichina*, *T. capitatus* and *T. vulgaris*). Data collected for antibacterial properties were analyzed by two-way analysis of variance to test the effects of two fixed factors: essential oil (levels: *T. zygis*, *T. mastichina*, *T. capitatus* and *T. vulgaris*) and bacterial strains (levels: *L. innocua*, *A. hydrophila*, *S. marcescens*, *A. faecalis*, *A. denitrificans*, *P. fragi*, *P. fluorescens*, *S. putrefaciens*, *E. amnigenus* and *E. gergoviae*). The Tukey’s post hoc test was applied for comparisons of means, differences were considered significant at *p* < 0.05. Statistical analysis and comparisons among means were carried out using the statistical package Statgraphics 5.1 for Windows (Statpoint Technologies Inc., Herndon, VA, USA).

## 3. Results and Discussion

### 3.1. Antioxidant Activity

The antioxidant activity of EOs obtained from *T. zygis*, *T. mastichina*, *T. capitatus* and *T. vulgaris* was determined using four different methodologies (DPPH and ABTS^•+^ scavenging activity, reducing power and chelating activity), due to the fact that a single method will provide basic information about antioxidant properties, but a combination of methods will describe the antioxidant properties of the sample in more detail [18]. The results are summarized in Table 1. With regard to DPPH assay, the EOs analyzed exhibited varying degrees of scavenging ability. *T. capitatus* EO showed the strongest (*p* < 0.05) radical scavenging effect, with an IC_50_ value of 0.60 mg/mL followed by *T. zygis* EO, which had an IC_50_ value of 0.90 mg/mL. *T. mastichina* and *T. vulgaris* EOs, in that order, showed the lowest scavenging activity (*p* < 0.05). In the case of ABTS^•+^ scavenging activity (Table 1), all EOs analyzed showed this ability. Again *T. capitatus* EO showed the lowest (*p* < 0.05) IC_50_ value, and therefore it had the greatest antioxidant activity. On the other hand, *T. vulgaris* EO had the lowest (*p* < 0.05) IC_50_ value. This strong radical scavenging potential capacity, measured with DPPH and ABTS assays, of the EOs analyzed could explained by the occurrence of hydroxylated compounds such as terpenoids in their composition [19]. 

Table 1 shows the ferric reducing antioxidant power obtained using the FRAP assay. *T. capitatus* EO had the highest (*p* < 0.05) ferric reducing capacity in terms of Trolox concentrations. It was followed by *T. zygis* EO. *T. mastichina* and *T. vulgaris* EOs had lower (*p* < 0.05) ferric reducing capacity compared with the other EOs. Ferrous ion, normally present in foods, is recognised as an effective pro-oxidant agent. EOs displayed the ability to chelate pro-oxidant metal ions, such as iron and copper, consequently avoiding free radical formation from these pro-oxidants. The Fe^+2^ chelating capacity of different *Thymus* EOs is shown in Table 1. *T. capitatus* and *T. zygis* EOs, showed the highest values (*p* < 0.05) for chelating Fe^+2^, with IC_50_ values of 4.44 and 4.95 mg/mL, respectively. Once more, *T. mastichina* and *T. vulgaris* EOs had the lowest capacity (*p* < 0.05) to act as chelating agents.

The antioxidant activities of essential oils obtained from several thyme varieties have been reported by several studies [20,21,22]. Therefore, Zouari et al. [20] investigated the antioxidant activity of *Thymus algeriensis* Boiss. et Reut EO, which grows wild in Tunisia. They reported that *T. algeriensis* EO was able to reduce the stable free radical DPPH with an IC_50_ of 0.8 mg/mL. Viuda-Martos et al. [4] analyzed the antioxidant activity of *Thymus vulgaris* EO cultivated in Egypt. These authors reported that this EO showed, in a DPPH assay, an IC_50_ of 4.50 mg/mL, while in the FIC assay the EC_50_ was 0.27 mg/mL. Ruiz-Navajas, et al. [6] reported IC_50_ values for *Thymus piperella* EO, in DPPH and FIC assays, of 9.30 and 425 mg/mL, respectively, while for *Thymus moroderi* EO the IC_50_ values were 90 and 6 mg/mL, respectively. In a similar study, Ali et al. [22] analyzed the antioxidant activity of EO obtained from *Thymus algeriensis*. These authors reported that the level of antioxidant activity estimated by the DPPH (IC_50_ = 4.31–9.23 mg/mL) and ABTS (11.69–28.23 μg Trolox Equivalent/mg) tests was moderate. Nikolić et al. [23] reported that *Thymus serpyllum* essential oil showed the highest DPPH radical scavenging activity (IC_50_: 0.96 μg/mL), followed by the oils of *T. algeriensis* (IC_50_: 1.64 μg/mL) and *T. vulgaris* (IC_50_: 4.80 μg/mL).

The difference in the antioxidant capacity measured with four different tests, between the four *Thymus* species analyzed in this work could be explained by the different mechanisms involved in each corresponding assay; each EO had different compounds in its composition with specific capacities to participate in those mechanisms. However, it should be borne in mind that, as mentioned by Viuda-Martos et al. [24], it is very difficult to attribute the antioxidant effect of an EO to one or a few main constituents, due to an EO always containing a mixture of different chemical compounds, meaning that their biological profiles are probably the result of a synergism of all molecules present in the EO. It is even possible that the activity of the main components is modulated by other minor molecules, as mentioned Bakkali et al. [25]. However, it should be borne in mind that the activities of EOs such as antioxidants depend not only on their structural features, but also on many other factors, such as concentration, temperature, light, type of substrate, and physical state of the system, as well as on microcomponents acting as pro-oxidants or synergists [26]. Anyway, the use of EOs or the individual isolated components obtained from them are new approaches for increasing their efficacy, taking advantage of their synergistic and additive effects [27]. 

### 3.2. Antibacterial Activity

The antibacterial activity of EOs obtained from *T. zygis*, *T. mastichina*, *T. capitatus* and *T. vulgaris* was determined by the application of disk diffusion against a panel of ten bacteria commonly associated with refrigerated foods, either as indicator of pathogenic microorganism or as spoilage microorganism, using medium extracts obtained from meat homogenates as culture. Table 2 shows the antibacterial activity of *Thymus* EOs using a minced beef extract as culture medium. All EOs studied showed growth inhibitory activity against all strains tested. All EOs tested showed the largest halos of inhibition against *A. faecalis* and *L. innocua* strains. For both bacteria all EOs were extremely active. In the case of *A. faecalis*, the *T. capitatus* EO showed the greatest inhibition halos (*p* < 0.05), followed by the *T. zygis*, *T. vulgaris* and *T. mastichina* Eos; while for *L. innocua*, the EO obtained from *T. zygis* had the greatest inhibition halos (*p* < 0.05), followed by *T. mastichina*, *T. capitatus* and *T. vulgaris* Eos, which showed no differences (*p* > 0.05) between them. For *E. amnigenus*, *E. gergoviae* and *P. fluorescens*, *T. zygis* and *T. capitatus* EOs showed the highest inhibitory activity on these strains, with no statistically significant differences (*p* > 0.05) between them. These EOs were moderately active against *Enterobacter* spp. and active on *P. fluorescens.* For *A. denitrifi*cans, *A. hydrophila*, *P. fragi*, *S. marcescens* and *S. putrefaciens*, *T. capitatus* EO showed the highest inhibition halos (*p* < 0.05), and their activity against these strains could be classified as active or extremely active. For these bacteria, the next most effective was the *T. zygis* EO, and its activity could be classified as moderately active or active.

With regard to the antibacterial activity of *Thymus* EOs using a cooked ham extract as culture medium (Table 3); again, all EOs analyzed had antibacterial activity on all bacteria strains tested. Except for *S. putrefaciens*, *T. vulgaris* EO showed the lowest inhibition zones (*p* < 0.05) of all EOs analyzed, and could be classified as not active or active depending of bacteria strain. On the other hand, *T. capitatus* EO showed the greatest (*p* < 0.05) inhibition halos against *A. denitrifi*cans, *A. faecalis*, *E. amnigenus* and *P. fluorescens*, while *T. zygis* EO had the highest inhibition halos (*p* < 0.05) against *L. innocua*, *A. hydrophila* and *P. fragi*. These EOs, against these strains, could be classified as moderately active or active. For *E. gergoviae* and *S. marcescens*, no statistical differences (*p* > 0.05) were found between *T. capitatus* and *T. zygis* EOs.

Table 4 shows the antibacterial activity of *T. capitatus*, *T. mastichina*, *T. vulgaris* and *T. zygis* EOs against several bacteria strains using a dry-cured sausage extract as culture medium. In this case, not all EOs studied showed growth inhibitory activity against all strains tested. Thus, for *E. gergoviae*, only *T. vulgaris* EOs produced inhibition halos while for *A. faecalis* and *E. amnigenus*, only *T. mastichina* and *T. vulgaris* EOs had antibacterial activity, with *T. vulgaris* EOs presenting higher (*p* < 0.05) inhibition halos. For *A. hydrophila*, *A. denitrificans*, *S. marcescens* and *S. putrefaciens* strains, the EOs obtained from *T. capitatus* and *T. zygis* showed the greatest inhibition halos, with no statistically significant differences (*p* > 0.05) between them. These EOs could be classified as moderately active against these bacteria, except on *A. hydrophila*. In this case, their activity is extremely active. In the case of *Pseudomonas* (*P. fragi* and *P. fluorescens*), *T. capitatus* EOs showed the greatest inhibition halos (*p* < 0.05), followed by *T. zygis*, *T. mastichina* and *T. vulgaris* EOs.

To our knowledge, there are no studies where the antibacterial activity of *Thymus* EOs were determined using extracts from meat homogenates as culture medium. However, the antibacterial activity of EOs obtained from *Thymus* species has been widely determined. Ruiz-Navajas et al. [28] reported that the EOs obtained from two *Thymus* species endemic of eastern of Spain, such as *Thymus moroderi* and *Thymus piperella*, are a source of important bioactive compounds with antibacterial capacities against several Gram-positive and Gram-negative bacteria. The inhibition zones of microbial strains were in the range of 16.0–45.00 mm. Likewise, Fatma et al. [29] investigated the antibacterial activity of *Thymus hirtus* sp. *Algeriensis* EO cultivated in Tunisia against six bacterial strains; namely, *Escherichia coli*, *Pseudomonas aeruginosa*, *Salmonella enteritidis*, *Staphylococcus aureus*, *Bacillus subtilis* and *Listeria monocytogenes*. These authors found that *T. hirtus* sp. *Algeriensis* EO was capable of inhibiting the growth of bacterial organisms tested with inhibition zones of between 9 and 65 mm. Similarly, Tepe et al. [30] analyzed the antibacterial potential of *Thymus hyemalis* EO against several bacterial strains, and they found that in the presence of this EO, no activity was observed against *Enterobacter aerogenes*, *Klebsiella pneumonia*, *P. aeruginosa*, *L. monocytogenes* and *P. fluorescens*. The most sensitive microorganisms were determined to be *Bacillus cereus* and *B. subtilis*, with an MIC value of 31.25 mg/mL, while *Enterococcus faecalis* and *S. aureus* had MIC values of 62.50 mg/mL. De Martino et al. [31] investigated the antibacterial properties of EOs obtained from *Thymus longicaulis* and *Thymus pulegioides*. These EOs showed important antibacterial activity against *Staphylococcus aureus*, *Streptococcus faecalis*, *Bacillus subtilis*, *Bacillus cereus*, *Proteus mirabilis*, *Enterobacter coli*, *Salmonella typhi Ty2* and *Pseudomonas aeruginosa*.

The antibacterial data obtained in this work showed that certain compounds in the oils were successfully antibacterial, augmenting the oils’ significance in inhibiting microbial pathogens [27]. Terpenes have been widely demonstrated as potent antimicrobial compounds, and their considerable contribution to the *Thymus* EOs composition could be responsible for the antibacterial activities achieved in the present study. Thus, some researchers have reported that there is a relationship between the chemical composition of the most abundant components in the EO and the antimicrobial activity [32]. For instance, the main components of the *Thymus* EOs analyzed in this work—thymol, carvacrol, linalool and 1,8-cineol—have been found in previous studies to exhibit strong antibacterial activity [33,34]. Nonetheless, as occurs with the antioxidant activity, the antibacterial properties showed by the *Thymus* EOs analyzed in the present study could result from a synergistic effect of more than one component. 

Additionally, it should be borne in mind, that when the EOs are used as antimicrobial agents in food systems, higher concentrations of EOs are needed in order to have comparable antimicrobial effects to those obtained in vitro. Factors present in complex food matrices such as fat content, proteins, water activity, pH, and enzymes can potentially diminish the efficacy of EOs [35]. Thus, high levels of fat and protein in food protect, to a certain extent, the bacteria strains from the action of EOs. Therefore, if the EOs are dissolved in the lipid phase of the food, it will be less available to act on the bacteria strains that are present in the aqueous phase. It is also important to highlight that, depending on the state of the fat fraction of food—either emulsified, degraded by action of the fermentation and curing process, or unaltered—the EOs may exert more or less activity against microbial growth, as is shown in this paper. 

Bacteria need water, several sources of nitrogen, and carbon, as well as numerous micronutrients. To have carbon and nitrogen sources such as mono- and disaccharides (as carbon sources) and small amino acids or peptides of molecular weight (as nitrogen sources) favors bacterial growth, and indeed is essential for some bacterial species. The extract of fresh meat has integrity and a low presence of simple sugar proteins or organic acids, and as such is less favorable as a substrate to bacterial growth that extracts cooked product (with dextrose added and caseinates or hydrolyzed soy protein as simple sources of carbon and nitrogen). Extract obtained from dry-cured product is a more favorable substrate for the growth of many bacteria, overcoming the low water activity of additional product due to the extraction, the extract contains dextrose and peptides because of the proteolytic activity of starter cultures. As mentioned above, the higher fat content of the dry-cured product extract can protect some bacteria from the effects of Eos, since they tend to be solubilized by the fat being less available to act on the bacteria. Therefore, EOs in fresh product extracts were more effective inhibitors of bacteria than in cooked and dry-cured extract products.

Although the EOs showed antibacterial properties, the reasons behind this ability are not well documented. For this reason, different mechanisms of action have been suggested. Thus, the available literature data show that the primary site of the toxic action of EOs is generally the plasmic membrane. Therefore, Horvathova et al. [36] mentioned that the effectiveness of EOs against several microorganisms is related to their hydrophobicity, which enables them to integrate into the lipids of the cell membrane and mitochondria, rendering them permeable and leading to leakage of cell contents. Gao et al. [37] reported that membrane function could be disturbed by inhibition of the proton motive force and electron transfer, or disruption of synthesis of critical macromolecules such as nucleic acid and protein. Arques et al. [38] proposed that the EOs affect microbial cells by various antimicrobial mechanisms, including disrupting enzyme systems, compromising the genetic material of bacteria, and forming fatty acid hydroperoxidase caused by oxygenation of unsaturated fatty acids. The essential oils might also inhibit the activity of protective enzymes and sequentially inhibit one or more biochemical pathways, as mentioned by Xing et al. [39]

## 4. Conclusions

The essential oils obtained from *Thymus vulgaris*, *Thymus mastichina* and mainly from *Thymus zygis* and *Thymus capitatus* may be used, by the food industry in general and the meat industry in particular, as potential natural or “green” additives to replace or reduce the use of chemical ones, since they show significant antioxidant and antibacterial properties. In addition, their efficacy as antibacterial agents have been demonstrated in model systems that closely simulate food composition. However, the use of these essential oils could be restricted by changes to the organoleptic properties of foods to which they are added. So, the most suitable essential oil must be chosen, at the right concentration, for each type of food.

## Figures and Tables

**Table 1:** Antioxidant activity of essential oils obtained from *T. capitatus*, *T. mastichina*, *T. vulgaris* and *T. zygis* determined using four different methods such as DPPH, ABTS, FRAP and FIC.

	DPPH Assay	ABTS Assay	FIC Assay	FRAP Assay
	IC_50_ (mg/mL)	IC_50_ (mg/mL)	IC_50_ (mg/mL)	(mg TE/mL)
*T. mastichina*	3.11 ± 0.11 ^b^	3.73 ± 0.14 ^b^	9.61 ± 0.19 ^b^	19.26 ± 0.10 ^c^
*T. zygis*	0.90 ± 0.03 ^c^	2.07 ± 0.06 ^c^	4.95 ± 0.14 ^c^	49.56 ± 0.09 ^b^
*T. vulgaris*	4.05 ± 0.09 ^a^	6.46 ± 0.11 ^a^	13.29 ± 0.18 ^a^	12.69 ± 0.03 ^d^
*T. capitatus*	0.60 ± 0.02 ^d^	1.41 ± 0.05 ^d^	4.44 ± 0.16 ^d^	58.12 ± 0.25 ^a^

DPPH: 2,2′-diphenyl-1-picrylhydrazyl Radical Scavenging Method; ABTS: Radical Cation (ABTS^•+^) Scavenging Activity Assay; FIC: Ferrous ion (Fe^2+^) chelating activity; FRAP: The ferric reducing antioxidant power. Values followed by the same lower-case letter within the same column are not significantly different (*p* > 0.05) according to Tukey’s Multiple Range Test.

**Table 2:** Antibacterial activity of *T. capitatus*, *T. mastichina*, *T. vulgaris* and *T. zygis* EOs against several bacterial strains using minced beef extract as culture medium.

	Diameter of Inhibition Zone (mm) Including Disc (9 mm)			
	*T. capitatus*	*T. mastichina*	*T. vulgaris*	*T. zygis*
*A. denitrificans*	28.37 ± 0.11 ^aD (++)^	11.29 ± 0.00 ^dE (−)^	19.82 ± 0.97 ^cB (+)^	23.92 ± 2.72 ^bD (++)^
*A. faecalis*	35.12 ± 0.30 ^aA (+++)^	16.91 ± 1.17 ^dB (+)^	32.73 ± 0.33 ^cA (+++)^	33.85 ± 0.36 ^bB (+++)^
*A. hydrophila*	30.69 ± 1.88 ^aC (+++)^	14.70 ± 0.98 ^dC (+)^	16.04 ± 0.11 ^cC (+)^	19.35 ± 0.07 ^bE (+)^
*E. amnigenus*	16.80 ± 0.28 ^aE (+)^	10.97 ± 0.01 ^cF (−)^	12.43 ± 0.18 ^b (+)^	17.98 ± 1.67 ^aE (+)^
*E. gergoviae*	13.78 ± 0.63 ^aF (+)^	10.82 ± 0.06 ^bF (−)^	10.77 ± 1.42 ^bE (−)^	13.92 ± 0.08 ^aF (+)^
*L. innocua*	34.07 ± 1.68 ^bA (+++)^	34.98 ± 1.67 ^bA (+++)^	33.77 ± 3.35 ^bA (+++)^	45.37 ± 5.98 ^aA (+++)^
*P. fluorescens*	29.36 ± 2.11 ^aCD (++)^	12.07 ± 1.10 ^cDE (+)^	19.91 ± 2.55 ^bB (+)^	27.72 ± 0.68 ^aC (++)^
*P. fragi*	29.80 ± 2.16 ^aCD (++)^	11.61 ± 0.01 ^dE (−)^	16.23 ± 2.29 ^cCD (+)^	26.61 ± 1.90 ^bC (++)^
*S. marcescens*	29.06 ± 1.10 ^aCD (++)^	11.84 ± 0.49 ^cE (−)^	16.84 ± 1.10 ^bCD (+)^	16.96 ± 0.05 ^bE (+)^
*S. putrefaciens*	32.04 ± 0.01 ^aB (+++)^	13.09 ± 0.40 ^dD (+)^	15.34 ± 0.00 ^cD (+)^	20.85 ± 3.28 ^bDE (+)^

For the same bacteria, values followed by the same lower-case letter within the same row are not significantly different (*p* > 0.05) according to Tukey’s Multiple Range Test. For the same essential oil, values followed by the same upper-case letter within the same column are not significantly different (*p* > 0.05) according to Tukey’s Multiple Range Test. Essential oils are classified as (−) not active, (+) moderately active, (++) active and (+++) extremely active.

**Table 3:** Antibacterial activity of *T. capitatus*, *T. mastichina*, *T. vulgaris* and *T. zygis* EOs against several bacterial strains using cooked ham extract as culture medium.

	Diameter of Inhibition Zone (mm) Including Disc (9 mm)			
	*T. capitatus*	*T. mastichina*	*T. vulgaris*	*T. zygis*
*A. denitrificans*	14.70 ± 0.14 ^aE (+)^	13.29 ± 0.61^bcB (+)^	13.59 ± 0.00 ^bE (+)^	13.11 ± 0.13 ^cE (+)^
*A. faecalis*	32.25 ± 0.01 ^aA (+++)^	15.34 ± 0.70 ^cA (+)^	24.55 ± 1.44 ^bA (++)^	25.39 ± 1.96 ^bA (++)^
*A. hydrophila*	14.49 ± 0.39 ^bE (+)^	12.13 ± 0.34 ^cC (+)^	14.74 ± 0.08 ^bD (+)^	18.51 ± 0.24 ^aB (+)^
*E. amnigenus*	16.29 ± 0.50 ^aC (+)^	10.69 ± 0.37^cE (−)^	10.87 ± 0.10 ^cF (−)^	15.08 ± 0.90 ^bD (+)^
*E. gergoviae*	16.51 ± 1.73 ^aC (+)^	13.81 ± 0.28 ^cB (+)^	14.46 ± 0.20 ^bDE (+)^	17.32 ± 0.97 ^aC (+)^
*L. innocua*	20.86 ± 0.59 ^bB (++)^	15.23 ± 1.22 ^cA (+)^	22.35 ± 0.40 ^aB (++)^	23.01 ± 0.78 ^aA (++)^
*P. fluorescens*	17.71 ± 0.85 ^aC (+)^	12.86 ± 0.56 ^dC (+)^	13.93 ± 0.45 ^cE (+)^	15.48 ± 0.25 ^bD (+)^
*P. fragi*	15.17 ± 0.15 ^bD (+)^	11.78 ± 0.06 ^dD (−)^	14.30 ± 0.64 ^cDE (+)^	16.68 ± 0.37 ^aC (+)^
*S. marcescens*	16.59 ± 1.09 ^aC (+)^	12.69 ± 0.01 ^bC (+)^	15.95 ± 0.80 ^aC (+)^	15.15 ± 0.00 ^aD (+)^
*S. putrefaciens*	13.75 ± 0.00 ^bF (+)^	14.34 ± 0.23 ^aA (+)^	9.77 ± 0.04 ^dG (−)^	12.98 ± 0.11 ^cE (+)^

For the same bacteria, values followed by the same lower-case letter within the same row are not significantly different (*p* > 0.05) according to Tukey’s Multiple Range Test. For the same essential oil, values followed by the same upper-case letter within the same column are not significantly different (*p* > 0.05) according to Tukey’s Multiple Range Test. Essential oils are classified as (−) not active, (+) moderately active, (++) active and (+++) extremely active.

**Table 4:** Antibacterial activity of *T. capitatus*, *T. mastechina*, *T. vulgaris* and *T. zygis* EOs against several bacterial strains using a dry-cured sausage extract as culture medium.

	Diameter of Inhibition Zone (mm) Including Disc (9 mm)			
	*T. capitatus*	*T. mastechina*	*T. vulgaris*	*T. zygis*
*A. denitrificans*	20.75 ± 1.57 ^aC (+)^	15.87 ± 1.10 ^bE (+)^	15.99 ± 1.03 ^b (+)^	20.63 ± 0.28 ^aC (+)^
*A. faecalis*	9.00 ± 0.00 ^cE (−)^	16.03 ± 0.30 ^bE (+)^	21.38 ± 2.08 ^a (++)^	9.00 ± 0.00 ^cF (−)^
*A. hydrophila*	38.18 ± 1.48 ^aA (+++)^	24.94 ± 0.11 ^cA (++)^	29.91 ± 5.12 ^b (++)^	40.72 ± 0.85 ^aB (+++)^
*E. amnigenus*	9.00 ± 0.00 ^cE (−)^	17.31 ± 0.29 ^bC (+)^	19.19 ± 1.08 ^a (+)^	9.00 ± 0.00 ^cF (+−)^
*E. gergoviae*	9.00 ± 0.00 ^bE (−)^	9.00 ± 0.00 ^bH (−)^	12.85 ± 0.44 ^a (+)^	9.00 ± 0.00 ^bF (−)^
*L. innocua*	38.59 ± 1.37 ^bA (+++)^	19.45 ± 1.03 ^dB (+)^	22.82 ± 0.45 ^c (++)^	50.97 ± 5.17 ^aA (+++)^
*P. fluorescens*	19.28 ± 0.15 ^aC (+)^	16.70 ± 0.33 ^cD (+)^	11.49 ± 0.19 ^d (−)^	18.99 ± 0.90 ^bD (+)^
*P. fragi*	30.11 ± 0.02 ^aB (+++)^	14.19 ± 0.06 ^cF (+)^	13.26 ± 0.66 ^d (+)^	18.49 ± 0.28 ^bD (+)^
*S. marcescens*	16.60 ± 0.33 ^aD (+)^	11.49 ± 0.02 ^cG (−)^	14.82 ± 0.76 ^b (+)^	16.64 ± 0.10 ^aE (+)^
*S. putrefaciens*	18.96 ± 0.88 ^aC (+)^	15.82 ± 0.08 ^bE (+)^	13.45 ± 1.66 ^c (+)^	18.87 ± 0.51 ^aD (+)^

For the same bacteria, values followed by the same lower-case letter within the same row are not significantly different (*p* > 0.05) according to Tukey’s Multiple Range Test. For the same essential oil, values followed by the same upper-case letter within the same column are not significantly different (*p* > 0.05) according to Tukey’s Multiple Range Test. Essential oils are classified as (−) not active, (+) moderately active, (++) active and (+++) extremely active.
